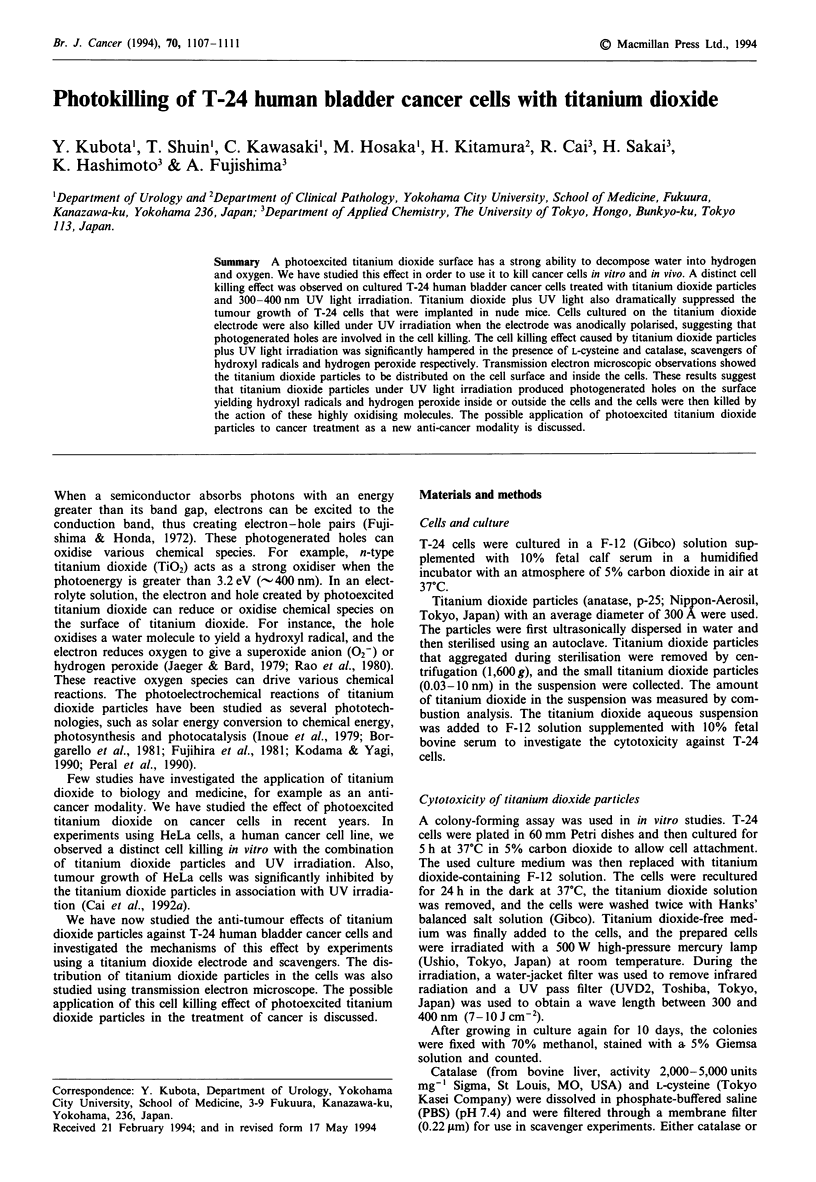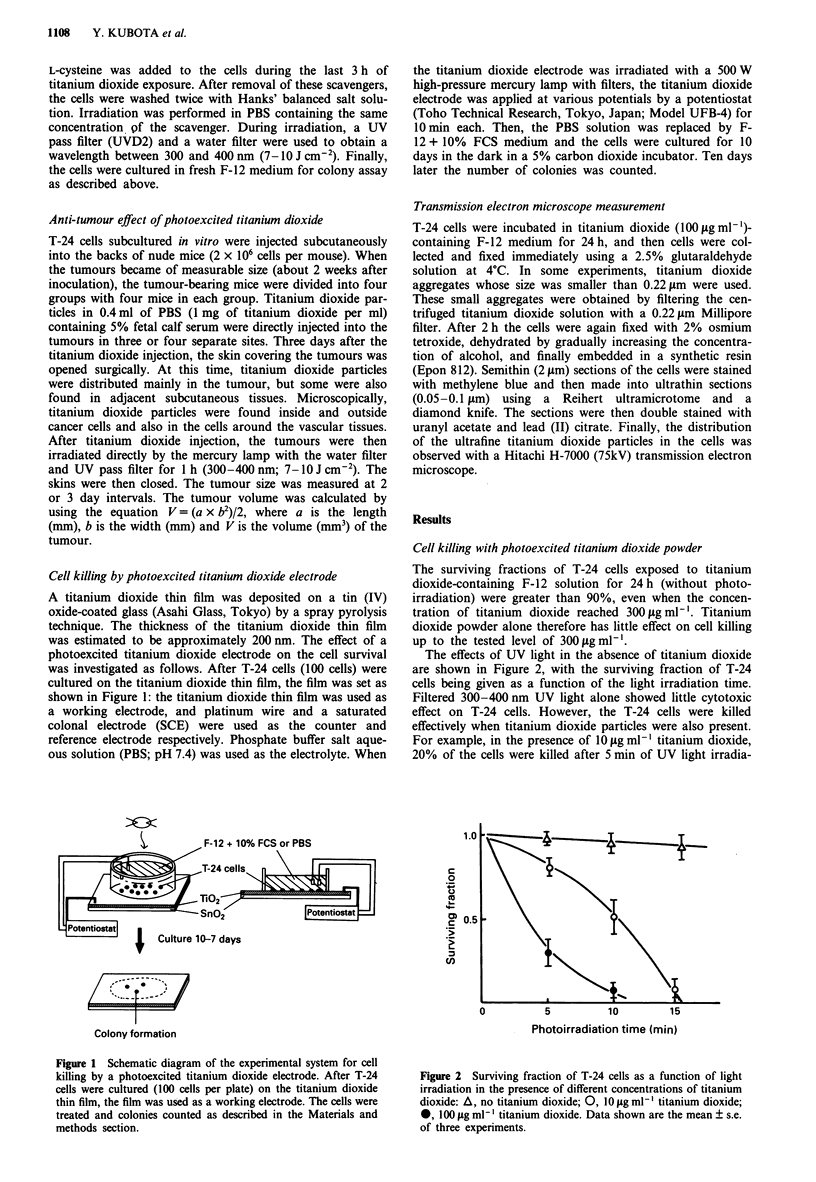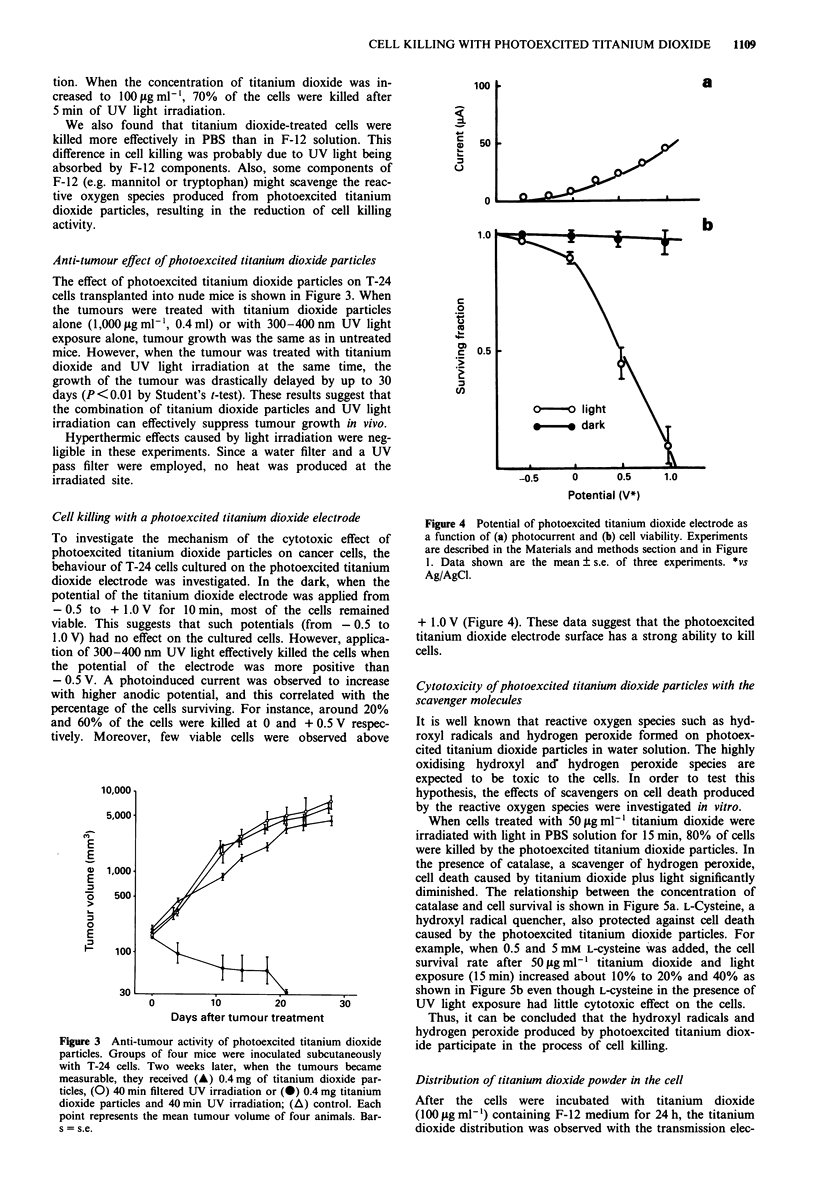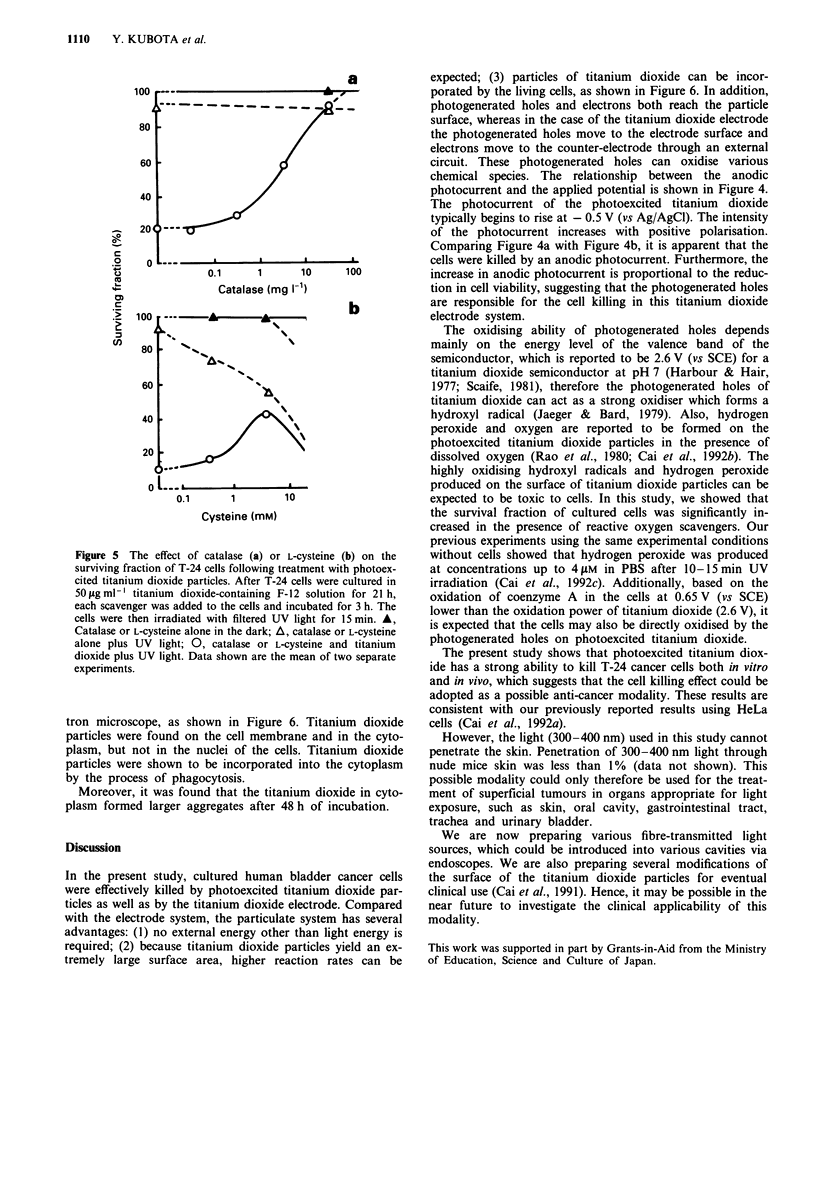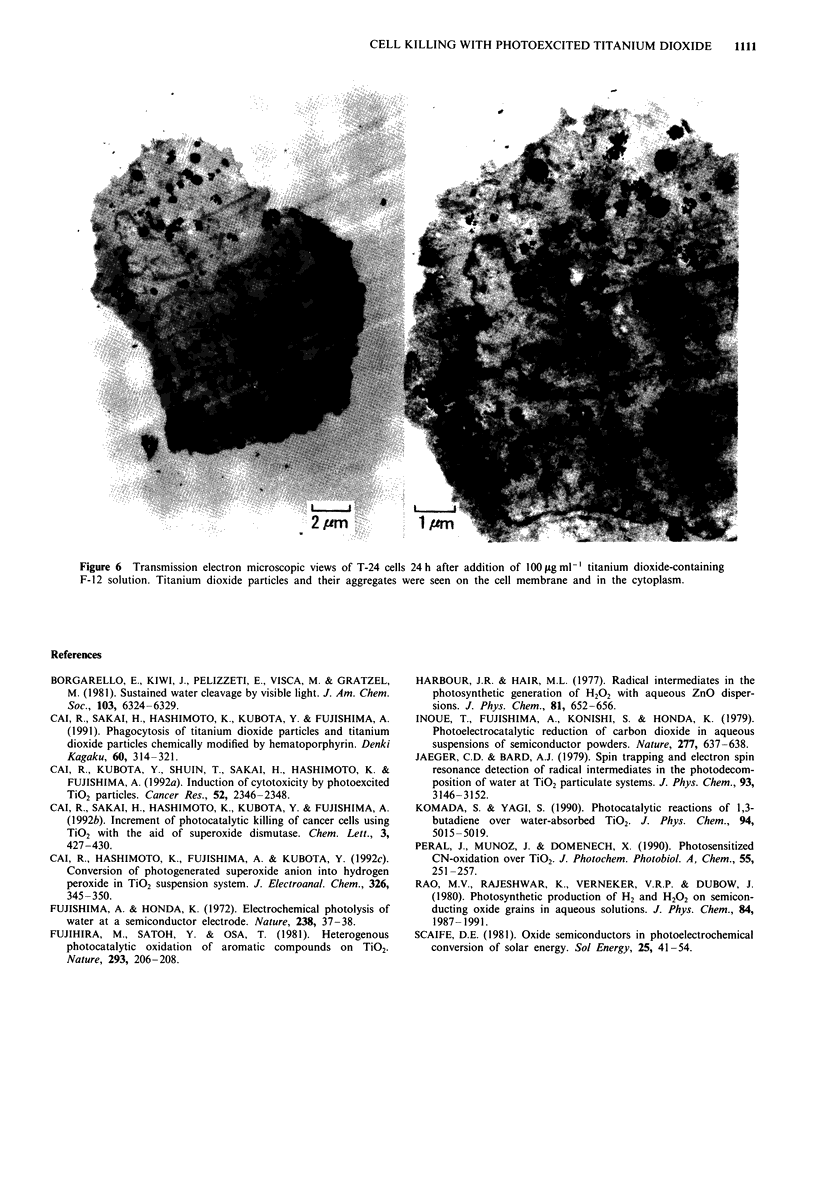# Photokilling of T-24 human bladder cancer cells with titanium dioxide.

**DOI:** 10.1038/bjc.1994.456

**Published:** 1994-12

**Authors:** Y. Kubota, T. Shuin, C. Kawasaki, M. Hosaka, H. Kitamura, R. Cai, H. Sakai, K. Hashimoto, A. Fujishima

**Affiliations:** Department of Urology, Yokohama City University, School of Medicine, Japan.

## Abstract

**Images:**


Br. J. Cancer (1994), 70, 1107–1111 © Macmillan Press Ltd., 1994

### Photokilling of T-24 human bladder cancer cells with titanium dioxide

Y. Kubota[1], T. Shuin[1], C. Kawasaki[1], M. Hosaka[1], H. Kitamura[2], R. Cai[3], H. Sakai[3], K. Hashimoto[3] & A. Fujishima[3]

[1]*Department of Urology and* [2]*Department of Clinical Pathology, Yokohama City University, School of Medicine, Fukuura, Kanazawa-ku, Yokohama 236, Japan;* [3]*Department of Applied Chemistry, The University of Tokyo, Hongo, Bunkyo-ku, Tokyo 113, Japan.*

**Summary** A photoexcited titanium dioxide surface has a strong ability to decompose water into hydrogen and oxygen. We have studied this effect in order to use it to kill cancer cells *in vitro* and *in vivo*. A distinct cell killing effect was observed on cultured T-24 human bladder cancer cells treated with titanium dioxide particles and 300–400 nm UV light irradiation. Titanium dioxide plus UV light also dramatically suppressed the tumour growth of T-24 cells that were implanted in nude mice. Cells cultured on the titanium dioxide electrode were also killed under UV irradiation when the electrode was anodically polarised, suggesting that photogenerated holes are involved in the cell killing. The cell killing effect caused by titanium dioxide particles plus UV light irradiation was significantly hampered in the presence of L-cysteine and catalase, scavengers of hydroxyl radicals and hydrogen peroxide respectively. Transmission electron microscopic observations showed the titanium dioxide particles to be distributed on the cell surface and inside the cells. These results suggest that titanium dioxide particles under UV light irradiation produced photogenerated holes on the surface yielding hydroxyl radicals and hydrogen peroxide inside or outside the cells and the cells were then killed by the action of these highly oxidising molecules. The possible application of photoexcited titanium dioxide particles to cancer treatment as a new anti-cancer modality is discussed.

When a semiconductor absorbs photons with an energy greater than its band gap, electrons can be excited to the conduction band, thus creating electron–hole pairs (Fujishima & Honda, 1972). These photogenerated holes can oxidise various chemical species. For example, *n*-type titanium dioxide ($TiO_2$) acts as a strong oxidiser when the photoenergy is greater than 3.2 eV ($\sim$400 nm). In an electrolyte solution, the electron and hole created by photoexcited titanium dioxide can reduce or oxidise chemical species on the surface of titanium dioxide. For instance, the hole oxidises a water molecule to yield a hydroxyl radical, and the electron reduces oxygen to give a superoxide anion ($O_2^-$) or hydrogen peroxide (Jaeger & Bard, 1979; Rao *et al.*, 1980). These reactive oxygen species can drive various chemical reactions. The photoelectrochemical reactions of titanium dioxide particles have been studied as several phototechnologies, such as solar energy conversion to chemical energy, photosynthesis and photocatalysis (Inoue *et al.*, 1979; Borgarello *et al.*, 1981; Fujihira *et al.*, 1981; Kodama & Yagi, 1990; Peral *et al.*, 1990).

Few studies have investigated the application of titanium dioxide to biology and medicine, for example as an anticancer modality. We have studied the effect of photoexcited titanium dioxide on cancer cells in recent years. In experiments using HeLa cells, a human cancer cell line, we observed a distinct cell killing *in vitro* with the combination of titanium dioxide particles and UV irradiation. Also, tumour growth of HeLa cells was significantly inhibited by the titanium dioxide particles in association with UV irradiation (Cai *et al.*, 1992*a*).

We have now studied the anti-tumour effects of titanium dioxide particles against T-24 human bladder cancer cells and investigated the mechanisms of this effect by experiments using a titanium dioxide electrode and scavengers. The distribution of titanium dioxide particles in the cells was also studied using transmission electron microscope. The possible application of this cell killing effect of photoexcited titanium dioxide particles in the treatment of cancer is discussed.

Correspondence: Y. Kubota, Department of Urology, Yokohama City University, School of Medicine, 3-9 Fukuura, Kanazawa-ku, Yokohama, 236, Japan.
Received 21 February 1994; and in revised form 17 May 1994

### Materials and methods

### *Cells and culture*

T-24 cells were cultured in a F-12 (Gibco) solution supplemented with 10% fetal calf serum in a humidified incubator with an atmosphere of 5% carbon dioxide in air at 37°C.

Titanium dioxide particles (anatase, p-25; Nippon-Aerosil, Tokyo, Japan) with an average diameter of 300 Å were used. The particles were first ultrasonically dispersed in water and then sterilised using an autoclave. Titanium dioxide particles that aggregated during sterilisation were removed by centrifugation (1,600 *g*), and the small titanium dioxide particles (0.03–10 nm) in the suspension were collected. The amount of titanium dioxide in the suspension was measured by combustion analysis. The titanium dioxide aqueous suspension was added to F-12 solution supplemented with 10% fetal bovine serum to investigate the cytotoxicity against T-24 cells.

### *Cytotoxicity of titanium dioxide particles*

A colony-forming assay was used in *in vitro* studies. T-24 cells were plated in 60 mm Petri dishes and then cultured for 5 h at 37°C in 5% carbon dioxide to allow cell attachment. The used culture medium was then replaced with titanium dioxide-containing F-12 solution. The cells were recultured for 24 h in the dark at 37°C, the titanium dioxide solution was removed, and the cells were washed twice with Hanks' balanced salt solution (Gibco). Titanium dioxide-free medium was finally added to the cells, and the prepared cells were irradiated with a 500 W high-pressure mercury lamp (Ushio, Tokyo, Japan) at room temperature. During the irradiation, a water-jacket filter was used to remove infrared radiation and a UV pass filter (UVD2, Toshiba, Tokyo, Japan) was used to obtain a wave length between 300 and 400 nm (7–10 J $cm^{-2}$).

After growing in culture again for 10 days, the colonies were fixed with 70% methanol, stained with a 5% Giemsa solution and counted.

Catalase (from bovine liver, activity 2,000–5,000 units $mg^{-1}$ Sigma, St Louis, MO, USA) and L-cysteine (Tokyo Kasei Company) were dissolved in phosphate-buffered saline (PBS) (pH 7.4) and were filtered through a membrane filter (0.22 μm) for use in scavenger experiments. Either catalase or

L-cysteine was added to the cells during the last 3 h of titanium dioxide exposure. After removal of these scavengers, the cells were washed twice with Hanks' balanced salt solution. Irradiation was performed in PBS containing the same concentration of the scavenger. During irradiation, a UV pass filter (UVD2) and a water filter were used to obtain a wavelength between 300 and 400 nm (7–10 J cm$^{-2}$). Finally, the cells were cultured in fresh F-12 medium for colony assay as described above.

*Anti-tumour effect of photoexcited titanium dioxide*

T-24 cells subcultured *in vitro* were injected subcutaneously into the backs of nude mice ($2 \times 10^6$ cells per mouse). When the tumours became of measurable size (about 2 weeks after inoculation), the tumour-bearing mice were divided into four groups with four mice in each group. Titanium dioxide particles in 0.4 ml of PBS (1 mg of titanium dioxide per ml) containing 5% fetal calf serum were directly injected into the tumours in three or four separate sites. Three days after the titanium dioxide injection, the skin covering the tumours was opened surgically. At this time, titanium dioxide particles were distributed mainly in the tumour, but some were also found in adjacent subcutaneous tissues. Microscopically, titanium dioxide particles were found inside and outside cancer cells and also in the cells around the vascular tissues. After titanium dioxide injection, the tumours were then irradiated directly by the mercury lamp with the water filter and UV pass filter for 1 h (300–400 nm; 7–10 J cm$^{-2}$). The skins were then closed. The tumour size was measured at 2 or 3 day intervals. The tumour volume was calculated by using the equation $V = (a \times b^2)/2$, where $a$ is the length (mm), $b$ is the width (mm) and $V$ is the volume (mm$^3$) of the tumour.

*Cell killing by photoexcited titanium dioxide electrode*

A titanium dioxide thin film was deposited on a tin (IV) oxide-coated glass (Asahi Glass, Tokyo) by a spray pyrolysis technique. The thickness of the titanium dioxide thin film was estimated to be approximately 200 nm. The effect of a photoexcited titanium dioxide electrode on the cell survival was investigated as follows. After T-24 cells (100 cells) were cultured on the titanium dioxide thin film, the film was set as shown in Figure 1: the titanium dioxide thin film was used as a working electrode, and platinum wire and a saturated colonal electrode (SCE) were used as the counter and reference electrode respectively. Phosphate buffer salt aqueous solution (PBS; pH 7.4) was used as the electrolyte. When the titanium dioxide electrode was irradiated with a 500 W high-pressure mercury lamp with filters, the titanium dioxide electrode was applied at various potentials by a potentiostat (Toho Technical Research, Tokyo, Japan; Model UFB-4) for 10 min each. Then, the PBS solution was replaced by F-12 + 10% FCS medium and the cells were cultured for 10 days in the dark in a 5% carbon dioxide incubator. Ten days later the number of colonies was counted.

*Transmission electron microscope measurement*

T-24 cells were incubated in titanium dioxide (100 μg ml$^{-1}$)-containing F-12 medium for 24 h, and then cells were collected and fixed immediately using a 2.5% glutaraldehyde solution at 4°C. In some experiments, titanium dioxide aggregates whose size was smaller than 0.22 μm were used. These small aggregates were obtained by filtering the centrifuged titanium dioxide solution with a 0.22 μm Millipore filter. After 2 h the cells were again fixed with 2% osmium tetroxide, dehydrated by gradually increasing the concentration of alcohol, and finally embedded in a synthetic resin (Epon 812). Semithin (2 μm) sections of the cells were stained with methylene blue and then made into ultrathin sections (0.05–0.1 μm) using a Reihert ultramicrotome and a diamond knife. The sections were then double stained with uranyl acetate and lead (II) citrate. Finally, the distribution of the ultrafine titanium dioxide particles in the cells was observed with a Hitachi H-7000 (75kV) transmission electron microscope.

### Results

*Cell killing with photoexcited titanium dioxide powder*

The surviving fractions of T-24 cells exposed to titanium dioxide-containing F-12 solution for 24 h (without photoirradiation) were greater than 90%, even when the concentration of titanium dioxide reached 300 μg ml$^{-1}$. Titanium dioxide powder alone therefore has little effect on cell killing up to the tested level of 300 μg ml$^{-1}$.

The effects of UV light in the absence of titanium dioxide are shown in Figure 2, with the surviving fraction of T-24 cells being given as a function of the light irradiation time. Filtered 300–400 nm UV light alone showed little cytotoxic effect on T-24 cells. However, the T-24 cells were killed effectively when titanium dioxide particles were also present. For example, in the presence of 10 μg ml$^{-1}$ titanium dioxide, 20% of the cells were killed after 5 min of UV light irradia-

**Figure 1** Schematic diagram of the experimental system for cell killing by a photoexcited titanium dioxide electrode. After T-24 cells were cultured (100 cells per plate) on the titanium dioxide thin film, the film was used as a working electrode. The cells were treated and colonies counted as described in the Materials and methods section.

**Figure 2** Surviving fraction of T-24 cells as a function of light irradiation in the presence of different concentrations of titanium dioxide: Δ, no titanium dioxide; O, 10 μg ml$^{-1}$ titanium dioxide; ●, 100 μg ml$^{-1}$ titanium dioxide. Data shown are the mean ± s.e. of three experiments.

tion. When the concentration of titanium dioxide was increased to 100 μg ml$^{-1}$, 70% of the cells were killed after 5 min of UV light irradiation.

We also found that titanium dioxide-treated cells were killed more effectively in PBS than in F-12 solution. This difference in cell killing was probably due to UV light being absorbed by F-12 components. Also, some components of F-12 (e.g. mannitol or tryptophan) might scavenge the reactive oxygen species produced from photoexcited titanium dioxide particles, resulting in the reduction of cell killing activity.

### *Anti-tumour effect of photoexcited titanium dioxide particles*

The effect of photoexcited titanium dioxide particles on T-24 cells transplanted into nude mice is shown in Figure 3. When the tumours were treated with titanium dioxide particles alone (1,000 μg ml$^{-1}$, 0.4 ml) or with 300–400 nm UV light exposure alone, tumour growth was the same as in untreated mice. However, when the tumour was treated with titanium dioxide and UV light irradiation at the same time, the growth of the tumour was drastically delayed by up to 30 days ($P<0.01$ by Student's *t*-test). These results suggest that the combination of titanium dioxide particles and UV light irradiation can effectively suppress tumour growth *in vivo*.

Hyperthermic effects caused by light irradiation were negligible in these experiments. Since a water filter and a UV pass filter were employed, no heat was produced at the irradiated site.

### *Cell killing with a photoexcited titanium dioxide electrode*

To investigate the mechanism of the cytotoxic effect of photoexcited titanium dioxide particles on cancer cells, the behaviour of T-24 cells cultured on the photoexcited titanium dioxide electrode was investigated. In the dark, when the potential of the titanium dioxide electrode was applied from − 0.5 to + 1.0 V for 10 min, most of the cells remained viable. This suggests that such potentials (from − 0.5 to 1.0 V) had no effect on the cultured cells. However, application of 300–400 nm UV light effectively killed the cells when the potential of the electrode was more positive than − 0.5 V. A photoinduced current was observed to increase with higher anodic potential, and this correlated with the percentage of the cells surviving. For instance, around 20% and 60% of the cells were killed at 0 and + 0.5 V respectively. Moreover, few viable cells were observed above

**Figure 3** Anti-tumour activity of photoexcited titanium dioxide particles. Groups of four mice were inoculated subcutaneously with T-24 cells. Two weeks later, when the tumours became measurable, they received (▲) 0.4 mg of titanium dioxide particles, (○) 40 min filtered UV irradiation or (●) 0.4 mg titanium dioxide particles and 40 min UV irradiation; (△) control. Each point represents the mean tumour volume of four animals. Bars = s.e.

**Figure 4** Potential of photoexcited titanium dioxide electrode as a function of (**a**) photocurrent and (**b**) cell viability. Experiments are described in the Materials and methods section and in Figure 1. Data shown are the mean ± s.e. of three experiments. **vs* Ag/AgCl.

+ 1.0 V (Figure 4). These data suggest that the photoexcited titanium dioxide electrode surface has a strong ability to kill cells.

### *Cytotoxicity of photoexcited titanium dioxide particles with the scavenger molecules*

It is well known that reactive oxygen species such as hydroxyl radicals and hydrogen peroxide formed on photoexcited titanium dioxide particles in water solution. The highly oxidising hydroxyl and hydrogen peroxide species are expected to be toxic to the cells. In order to test this hypothesis, the effects of scavengers on cell death produced by the reactive oxygen species were investigated *in vitro*.

When cells treated with 50 μg ml$^{-1}$ titanium dioxide were irradiated with light in PBS solution for 15 min, 80% of cells were killed by the photoexcited titanium dioxide particles. In the presence of catalase, a scavenger of hydrogen peroxide, cell death caused by titanium dioxide plus light significantly diminished. The relationship between the concentration of catalase and cell survival is shown in Figure 5a. L-Cysteine, a hydroxyl radical quencher, also protected against cell death caused by the photoexcited titanium dioxide particles. For example, when 0.5 and 5 mM L-cysteine was added, the cell survival rate after 50 μg ml$^{-1}$ titanium dioxide and light exposure (15 min) increased about 10% to 20% and 40% as shown in Figure 5b even though L-cysteine in the presence of UV light exposure had little cytotoxic effect on the cells.

Thus, it can be concluded that the hydroxyl radicals and hydrogen peroxide produced by photoexcited titanium dioxide participate in the process of cell killing.

### *Distribution of titanium dioxide powder in the cell*

After the cells were incubated with titanium dioxide (100 μg ml$^{-1}$) containing F-12 medium for 24 h, the titanium dioxide distribution was observed with the transmission elec-

**Figure 5** The effect of catalase (**a**) or L-cysteine (**b**) on the surviving fraction of T-24 cells following treatment with photoexcited titanium dioxide particles. After T-24 cells were cultured in 50 μg ml$^{-1}$ titanium dioxide-containing F-12 solution for 21 h, each scavenger was added to the cells and incubated for 3 h. The cells were then irradiated with filtered UV light for 15 min. ▲, Catalase or L-cysteine alone in the dark; △, catalase or L-cysteine alone plus UV light; ○, catalase or L-cysteine and titanium dioxide plus UV light. Data shown are the mean of two separate experiments.

tron microscope, as shown in Figure 6. Titanium dioxide particles were found on the cell membrane and in the cytoplasm, but not in the nuclei of the cells. Titanium dioxide particles were shown to be incorporated into the cytoplasm by the process of phagocytosis.

Moreover, it was found that the titanium dioxide in cytoplasm formed larger aggregates after 48 h of incubation.

### Discussion

In the present study, cultured human bladder cancer cells were effectively killed by photoexcited titanium dioxide particles as well as by the titanium dioxide electrode. Compared with the electrode system, the particulate system has several advantages: (1) no external energy other than light energy is required; (2) because titanium dioxide particles yield an extremely large surface area, higher reaction rates can be expected; (3) particles of titanium dioxide can be incorporated by the living cells, as shown in Figure 6. In addition, photogenerated holes and electrons both reach the particle surface, whereas in the case of the titanium dioxide electrode the photogenerated holes move to the electrode surface and electrons move to the counter-electrode through an external circuit. These photogenerated holes can oxidise various chemical species. The relationship between the anodic photocurrent and the applied potential is shown in Figure 4. The photocurrent of the photoexcited titanium dioxide typically begins to rise at − 0.5 V (*vs* Ag/AgCl). The intensity of the photocurrent increases with positive polarisation. Comparing Figure 4a with Figure 4b, it is apparent that the cells were killed by an anodic photocurrent. Furthermore, the increase in anodic photocurrent is proportional to the reduction in cell viability, suggesting that the photogenerated holes are responsible for the cell killing in this titanium dioxide electrode system.

The oxidising ability of photogenerated holes depends mainly on the energy level of the valence band of the semiconductor, which is reported to be 2.6 V (*vs* SCE) for a titanium dioxide semiconductor at pH 7 (Harbour & Hair, 1977; Scaife, 1981), therefore the photogenerated holes of titanium dioxide can act as a strong oxidiser which forms a hydroxyl radical (Jaeger & Bard, 1979). Also, hydrogen peroxide and oxygen are reported to be formed on the photoexcited titanium dioxide particles in the presence of dissolved oxygen (Rao *et al.*, 1980; Cai *et al.*, 1992*b*). The highly oxidising hydroxyl radicals and hydrogen peroxide produced on the surface of titanium dioxide particles can be expected to be toxic to cells. In this study, we showed that the survival fraction of cultured cells was significantly increased in the presence of reactive oxygen scavengers. Our previous experiments using the same experimental conditions without cells showed that hydrogen peroxide was produced at concentrations up to 4 μM in PBS after 10–15 min UV irradiation (Cai *et al.*, 1992*c*). Additionally, based on the oxidation of coenzyme A in the cells at 0.65 V (*vs* SCE) lower than the oxidation power of titanium dioxide (2.6 V), it is expected that the cells may also be directly oxidised by the photogenerated holes on photoexcited titanium dioxide.

The present study shows that photoexcited titanium dioxide has a strong ability to kill T-24 cancer cells both *in vitro* and *in vivo*, which suggests that the cell killing effect could be adopted as a possible anti-cancer modality. These results are consistent with our previously reported results using HeLa cells (Cai *et al.*, 1992*a*).

However, the light (300–400 nm) used in this study cannot penetrate the skin. Penetration of 300–400 nm light through nude mice skin was less than 1% (data not shown). This possible modality could only therefore be used for the treatment of superficial tumours in organs appropriate for light exposure, such as skin, oral cavity, gastrointestinal tract, trachea and urinary bladder.

We are now preparing various fibre-transmitted light sources, which could be introduced into various cavities via endoscopes. We are also preparing several modifications of the surface of the titanium dioxide particles for eventual clinical use (Cai *et al.*, 1991). Hence, it may be possible in the near future to investigate the clinical applicability of this modality.

This work was supported in part by Grants-in-Aid from the Ministry of Education, Science and Culture of Japan.

**Figure 6** Transmission electron microscopic views of T-24 cells 24 h after addition of 100 μg $ml^{-1}$ titanium dioxide-containing F-12 solution. Titanium dioxide particles and their aggregates were seen on the cell membrane and in the cytoplasm.

### References

BORGARELLO, E., KIWI, J., PELIZZETI, E., VISCA, M. & GRATZEL, M. (1981). Sustained water cleavage by visible light. *J. Am. Chem. Soc.*, **103**, 6324–6329.

CAI, R., SAKAI, H., HASHIMOTO, K., KUBOTA, Y. & FUJISHIMA, A. (1991). Phagocytosis of titanium dioxide particles and titanium dioxide particles chemically modified by hematoporphyrin. *Denki Kagaku*, **60**, 314–321.

CAI, R., KUBOTA, Y., SHUIN, T., SAKAI, H., HASHIMOTO, K. & FUJISHIMA, A. (1992*a*). Induction of cytotoxicity by photoexcited $TiO_2$ particles. *Cancer Res.*, **52**, 2346–2348.

CAI, R., SAKAI, H., HASHIMOTO, K., KUBOTA, Y. & FUJISHIMA, A. (1992*b*). Increment of photocatalytic killing of cancer cells using $TiO_2$ with the aid of superoxide dismutase. *Chem. Lett.*, **3**, 427–430.

CAI, R., HASHIMOTO, K., FUJISHIMA, A. & KUBOTA, Y. (1992*c*). Conversion of photogenerated superoxide anion into hydrogen peroxide in $TiO_2$ suspension system. *J. Electroanal. Chem.*, **326**, 345–350.

FUJISHIMA, A. & HONDA, K. (1972). Electrochemical photolysis of water at a semiconductor electrode. *Nature*, **238**, 37–38.

FUJIHIRA, M., SATOH, Y. & OSA, T. (1981). Heterogenous photocatalytic oxidation of aromatic compounds on $TiO_2$. *Nature*, **293**, 206–208.

HARBOUR, J.R. & HAIR, M.L. (1977). Radical intermediates in the photosynthetic generation of $H_2O_2$ with aqueous ZnO dispersions. *J. Phys. Chem.*, **81**, 652–656.

INOUE, T., FUJISHIMA, A., KONISHI, S. & HONDA, K. (1979). Photoelectrocatalytic reduction of carbon dioxide in aqueous suspensions of semiconductor powders. *Nature*, **277**, 637–638.

JAEGER, C.D. & BARD, A.J. (1979). Spin trapping and electron spin resonance detection of radical intermediates in the photodecomposition of water at $TiO_2$ particulate systems. *J. Phys. Chem.*, **93**, 3146–3152.

KOMADA, S. & YAGI, S. (1990). Photocatalytic reactions of 1,3-butadiene over water-absorbed $TiO_2$. *J. Phys. Chem.*, **94**, 5015–5019.

PERAL, J., MUNOZ, J. & DOMENECH, X. (1990). Photosensitized CN-oxidation over $TiO_2$. *J. Photochem. Photobiol. A, Chem.*, **55**, 251–257.

RAO, M.V., RAJESHWAR, K., VERNEKER, V.R.P. & DUBOW, J. (1980). Photosynthetic production of $H_2$ and $H_2O_2$ on semiconducting oxide grains in aqueous solutions. *J. Phys. Chem.*, **84**, 1987–1991.

SCAIFE, D.E. (1981). Oxide semiconductors in photoelectrochemical conversion of solar energy. *Sol Energy*, **25**, 41–54.